# An Algorithm That Identifies Coronary and Heart Failure Events in the Electronic Health Record

**DOI:** 10.5888/pcd10.120097

**Published:** 2013-02-28

**Authors:** Thomas E. Kottke, Courtney Jordan Baechler

**Affiliations:** Author Information: Courtney Jordan Baechler, Department of Medicine, School of Public Health, University of Minnesota, Minneapolis, Minnesota.

## Abstract

**Introduction:**

The advent of universal health care coverage in the United States and the use of electronic health records can make the medical record a disease surveillance tool. The objective of our study was to identify criteria that accurately categorize acute coronary and heart failure events by using electronic health record data exclusively so that the medical record can be used for surveillance without manual record review.

**Methods:**

We serially compared 3 computer algorithms to manual record review. The first 2 algorithms relied on ICD-9-CM (*International Classification of Diseases, 9th Revision, Clinical Modification*) codes, troponin levels, electrocardiogram (ECG) data, and echocardiograph data. The third algorithm relied on a detailed coding system, Intelligent Medical Objects, Inc., (IMO) interface terminology, troponin levels, and echocardiograph data.

**Results:**

Cohen’s κ for the initial algorithm was 0.47 (95% confidence interval [CI], 0.41–0.54). Cohen’s κ was 0.61 (95% CI, 0.55–0.68) for the second algorithm. Cohen’s κ for the third algorithm was 0.99 (95% CI, 0.98–1.00).

**Conclusion:**

Electronic medical record data are sufficient to categorize coronary heart disease and heart failure events without manual record review. However, only moderate agreement with medical record review can be achieved when the classification is based on 4-digit ICD-9-CM codes because ICD-9-CM 410.9 includes myocardial infarction with elevation of the ST segment on ECG (STEMI) and myocardial infarction without elevation of the ST segment on ECG (nSTEMI). Nearly perfect agreement can be achieved using IMO interface terminology, a more detailed coding system that tracks to ICD9, ICD10 (*International Classification of Diseases, Tenth Revision, Clinical Modification*), and SnoMED-CT (Systematized Nomenclature of Medicine – Clinical Terms).

## MEDSCAPE CME

Medscape, LLC is pleased to provide online continuing medical education (CME) for this journal article, allowing clinicians the opportunity to earn CME credit.

This activity has been planned and implemented in accordance with the Essential Areas and policies of the Accreditation Council for Continuing Medical Education through the joint sponsorship of Medscape, LLC and Preventing Chronic Disease. Medscape, LLC is accredited by the ACCME to provide continuing medical education for physicians.

Medscape, LLC designates this Journal-based CME activity for a maximum of 1 **AMA PRA Category 1 Credit(s)™**. Physicians should claim only the credit commensurate with the extent of their participation in the activity.

All other clinicians completing this activity will be issued a certificate of participation. To participate in this journal CME activity: (1) review the learning objectives and author disclosures; (2) study the education content; (3) take the post-test with a 70% minimum passing score and complete the evaluation at www.medscape.org/journal/pcd (4) view/print certificate.

**Release date: February 27, 2013; Expiration date: February 27, 2014**

### Learning Objectives

Upon completion of this activity, participants will be able to:

- Analyze recommendations regarding surveillance systems for cardiovascular disease
- Compare electronic with manual health records in identifying cases of cardiovascular disease
- Assess the accuracy of electronic electrocardiogram data in identifying cases of myocardial infarction
- Distinguish strategies to improve the accuracy of electronic records in the surveillance of cardiovascular disease

**EDITORS**

Rosemarie Perrin, editor; Caran Wilbanks, editor, *Preventing Chronic Disease*. Disclosure: Rosemarie Perrin and Caran Wilbanks have disclosed no relevant financial relationships.

**CME AUTHOR**

Charles P. Vega, MD, Health Sciences Clinical Professor; Residency Director, Department of Family Medicine, University of California, Irvine. Disclosure: Charles P. Vega, MD, has disclosed no relevant financial relationships.

**AUTHORS AND CREDENTIALS**

Disclosures: Thomas E. Kottke, MD, MSPH; and Courtney Jordan Baechler, MD, MCE, have disclosed no relevant financial relationships.

Affiliations: Thomas E. Kottke, HealthPartners Institute for Education and Research, Minneapolis, Minnesota; Courtney Jordan Baechler, Department of Medicine, School of Public Health, University of Minnesota, Minneapolis, Minnesota.

## Introduction

Surveillance to track the incidence, prevalence, and treatment of disease is a basic function of public health. Medical records can be used as surveillance tools, and the expense of manual record review could be avoided if medical records were representative of populations and if data in the medical record were standardized. Because the Patient Protection and Affordable Care Act will provide universal health care coverage for Americans (1), medical records could become a continuous population census. The Health Information Technology for Economic and Clinical Health (HITECH) Act and other authorities give the US Department of Health and Human Services the authority to promote electronic health records that permit electronic exchange of health information (2). Electronic information exchange could enable creation of a standardized data set for use in public health surveillance. The cataloging of cardiovascular and pulmonary disease surveys by an Institute of Medicine committee did not identify any algorithms that would enable use of the medical record to assess opportunities to improve outcomes across the broad spectrum of heart disease prevention and heart disease treatment (3). Nevertheless, the committee did recognize that electronic health records may be an emerging source of disease surveillance data and recommended that surveillance systems be designed so that they contribute to improved patient outcomes (3).

We are developing a decision-support tool that will identify opportunities for preventing heart disease and for improving outcomes for patients with heart disease (4–6). The first step in using the decision support tool is to classify each event by using an algorithm. In an attempt to eliminate the need for manual review of the medical record, we tested whether an electronic medical record of a medical group contained the data necessary to create an algorithm that could accurately categorize acute coronary heart disease and heart failure events.

## Methods

The first iteration of the computer algorithm was approved on November 20, 2008, by the HealthPartners Research Foundation Institutional Review Board as protocol no. 08–093, and the next 2 iterations were approved on September 21, 2009, as part of protocol no. 09–023. We completed the analysis on February 29, 2012.

### Manual record review criteria

The algorithm classifies coronary heart disease and heart failure events into 6 types: 1) acute myocardial infarction with ST segment elevation (STEMI) on electrocardiogram (ECG), 2) acute heart failure with a left ventricular ejection fraction at or below 35% (systolic heart failure), 3) acute myocardial infarction without ST segment elevation (nSTEMI) on ECG, 4) unstable angina or other acute ischemic event (unstable angina), 5) incident diagnosis made in the ambulatory setting (ambulatory presentation), and 6) chronic prevalent disease without an acute event during the period of interest (chronic disease). The classification system is hierarchical: a patient with a STEMI complicated by acute heart failure would be classified as having a STEMI. Likewise, a patient with acute heart failure, elevated levels of troponin (a protein that is present in blood during acute myocardial infarction), and an ECG that did not show a STEMI would be classified as having acute heart failure. The diagnoses are presented in hierarchical order in the first column of the Table.

**Table:** Manual Review Criteria and Criteria Used to Categorize Event Types: A Computer Algorithm That Identifies Coronary and Heart Failure Events in the Electronic Health Record^a^

Event Type	Manual Review Criteria	First Iteration of Computer Algorithm (N = 254)	Second Iteration of Computer Algorithm (N = 245)	Third Iteration of Computer Algorithm (N = 184)
1. STEMI	Hospital discharge code^b^ 410.0–410.6 or 410.8 OR 410.7 or 410.9 and ≥1 troponin level >3 × ULN and serial ECGs show evolving ST elevation by visual inspection and clinical history consistent with acute coronary syndrome OR diagnosis of STEMI on discharge summary.	Hospital discharge code 410.0–410.9 plus ≥1 troponin level > ULN; statements on ≥1 ECG indicate acute ST elevation consistent with acute MI; n = 64 when combined with nSTEMI category.	Hospital discharge code 410.0–410.6 or 410.8 OR code 410.7, 410.9, and any MUSE^c^ acronym 964–968 on ≥1 ECG plus ≥1 troponin level >3 × ULN; n = 24.	Any hospital discharge code starting with 410.0–410.6, 410.8, or IMO term 410.90CP, 410.90CS, 410.90CU, 410.90CX, 410.90DV, 410.90FZ, 410.90GB, 410.90GD, 410.91D, 410.92F, or 410.92S; n = 50.
2. Acute heart failure with symptomatic depressed left ventricular contractility	Hospital discharge code 425 or 428 as primary diagnosis and EF is ≤35%, BNP > ULN if measured, and dyspnea, pleural effusions, or other symptoms and signs are reason for hospitalization.	Hospital discharge code 425 or 428 as primary diagnosis plus BNP > ULN. Troponin may be positive; n = 56.	Hospital discharge code 425 or 428 as primary diagnosis plus BNP > ULN and EF ≤35%. Troponin may be positive; n = 21.	Hospital discharge code 425 or 428 as primary diagnosis plus BNP > ULN and EF ≤35%. Troponin may be positive; n = 34.
3. nSTEMI	410.7 or 410.9 or 420–429 and ≥1 troponin level >3 × ULN; and clinical history consistent with acute coronary syndrome. Serial ECGs do not show evolving ST elevation by visual inspection OR diagnosis of nSTEMI on discharge summary.	410.7–410.9 or 420–429 plus ≥1 troponin level > ULN and no ECG codes with evolving ST elevation; n = 64 when combined with STEMI category.	Hospital discharge code 410.7 or 410.9 without any MUSE^c^ acronym 964–968 on any ECG associated with the episode of care. Also, have ≥1 troponin level >3 × ULN; n = 50.	Any hospital discharge code starting with 410.7, or IMO^d^ term 410.90BT, 410.90CQ, 410.90CR, 410.90FY, 410.90GC, 410.90GE, 410.90N, 410.92E, 410.92H, or 410.91 (except 410.91D); n = 50.
4. Unstable angina	Hospitalized with code 411.x or 786.5 and clinical history consistent with heart disease problem, and no troponin level >3 × ULN.	Code 411.x and no troponin level > ULN; n = 70.	Hospital discharge code 411.x or 786.5x and no troponin level >3 × ULN. No procedure codes for insertion of pacemaker or defibrillator (37.80–37.99; CPT-4 33216); n = 50.	Category deleted by authors
5. Ambulatory presentation	Clinical history documents first episode of heart disease^e^ and presentation in an outpatient office.	No nonelective hospitalization at time of diagnosis. ≥2 years of observation before first diagnosis; n = 39.	First claim for heart disease^e^ is from an office. No prior claims for heart disease in the medical record. ≥2 years of observation before period of interest; n = 50.	Patient has heart disease diagnosis at any time. No nonelective hospitalization for heart disease^e^ during period of interest; n = 50.
6. Chronic prevalent disease	Heart disease^e^ present before the period of interest. No hospitalization for heart disease^e^ during the period of interest.	≥2 years of observation with codes 412–414 or 420–429 in first year of enrollment; n = 25.	≥2 years of observation with heart disease^e^ diagnosed before the period of interest. No hospitalization for heart disease^e^ or chest pain (786.5) during period of interest; n = 50.	
Cohen’s κ (95% confidence interval)	NA	0.47 (0.41–0.54)	0.61 (0.55–0.68)	0.99 (0.98–1.00)

Abbreviations: STEMI, myocardial infarction with ST elevation; ULN, upper limits of normal; ECG, electrocardiogram; MI, myocardial infarction; nSTEMI, myocardial infarction without ST elevation; EF, ejection fraction; BNP, B-type naturetic peptide; CPT, common procedural terminology; NA, not applicable.

a Categories are hierarchical with 1 being the highest. The number of cases identified by the computer algorithm is provided in each cell.

b All codes are from *International Classification of Diseases, 9th Revision, Clinical Modification* (14).

c MUSE Cardiology Information System (http://www.gehealthcare.com/euen/cardiology/products/diagnostic_ecg/database-management/index.html).

d Intelligent Medical Objects, Inc (http://www.e-imo.com/press/10960.aspx).

e Codes 410–414, or code 425 or code 428.

### First iteration

For the first iteration of the computer algorithm we used medical record data from 254 HealthPartners Medical Group (HPMG) patients who were also insured by HealthPartners. These patients were aged 40 to 74 years and had been treated for coronary heart disease or systolic heart failure from August 8, 2007, through July 31, 2008. August 8 was chosen as the start date because that was the day that the hospital ECG data file and the outpatient ECG data file were combined into a single file. To be included, patients needed to be HPMG patients so that we had access to their clinical records, and they needed to be insured by HealthPartners so that we had access to the ICD-9-CM diagnostic codes associated with their claims data. We used the computer algorithm to select patient records of coronary heart disease and heart failure events from each of the 6 categories. Each of the records was randomly assigned to a cardiologist (T.E.K. or C.J.B.) for manual record review, and 48 records were reviewed by both cardiologists. The cardiologists agreed on the diagnosis for 47 of the 48 patients. We sampled patient records until we had at least 30 verified cases of coronary heart disease and heart failure in each diagnostic category or had exhausted potential records for analysis. This process resulted in more than 30 cases identified as fitting some of the 6 diagnostic categories. The initial computer algorithm is presented in column 3 of the Table. We used SAS version 9.2 (SAS Institute Inc., Cary, North Carolina) to calculate Cohen’s κ for all events combined and revised the computer algorithm in response to post hoc analyses of the discordant cases.

After an ECG is interpreted by a physician, the text interpretation is converted to numerical codes and stored in an electronic database. During the first iteration, it was not possible to search the database for ECGs that had codes consistent with a STEMI. Software that supported search for codes became available when we were working on the second iteration, so we added ECG computer codes to the algorithms for STEMIs and nSTEMIs. As described in column 4 of the Table, we added ICD-9-CM 786.5 to the screening codes for unstable angina and excluded patients who had been admitted for pacemaker or defibrillator placement or revision. For the diagnosis of ambulatory presentation we required that the first insurance claim for heart disease was from an office visit, that there were no prior insurance claims for heart disease, and that there were 2 or more years of observations in the medical record. For chronic prevalent disease we required 2 or more years of observation with heart disease diagnosed before the period of interest and no hospitalization for heart disease or chest pain during the period of interest.

### Second iteration

We analyzed 245 cases to test the second iteration of the computer algorithm. We based the second iteration on data from HPMG patients who were also insured by HealthPartners and were treated by HPMG from January 1 through December 31, 2010. We used the second iteration to draw random samples for each diagnostic category, and 2 nurses and a physician reviewed the medical records. As with the first iteration, we calculated Cohen’s κ and revised the computer algorithm in response to post hoc analysis of the discordant cases.

### Third iteration

We analyzed 184 records for the third algorithm, 50 each for STEMI, nSTEMI, and chronic prevalent disease and 34 for acute heart failure with a depressed ejection fraction. We did not require HPMG patients to be insured by HealthPartners for the third iteration, because we did not need claims data to identify ICD-9-CM codes. However, 87% of the patients in the sampling frame had been insured by HealthPartners at some time, and 57% were insured by HealthPartners in 2010. As with the second iteration, the sampling time frame was from January 1 through December 31, 2010. We modified the computer algorithm for the third iteration (Table) with 3 major changes. First, we used Intelligent Medical Objects, Inc, interface terminology (IMO terms) to distinguish between STEMI and nSTEMI. IMO terms are codes that are based on ICD-9-CM codes but have specificity added with a fifth numerical digit and 1 or more letters following the numerals. For example, in addition to IMO terms 410.0–410.6 and 410.8 that are specific for STEMI, there are eleven 410.9 IMO terms that are specific for STEMI. In addition to 410.7, which is specific for nSTEMI there are at least 10 additional IMO terms that identify nSTEMI. IMO terms map to ICD-9-CM, ICD-10, and SnoMED-CT (7). Epic (Epic, Verona, Wisconsin), Cerner (Cerner Corporation, North Kansas City, Missouri), NextGen (NextGen Healthcare, Horsham, Pennsylvania), and several other electronic medical record systems incorporate IMO terms in their software. Epic stores these statements in the medical record.

We made the second modification, eliminating the category of unstable angina, because we found that most patients in this category already have a diagnosis of coronary heart disease. Because there is no compelling evidence that a patient hospitalized for chest pain with normal troponin levels benefits from an angiogram or therapies other than risk factor control, all gaps in their care would be detected during the analysis of patients with chronic disease.

We made the third modification, combining ambulatory presentation and chronic disease into a single category, because pursuit of therapies other than symptom control, daily aspirin, and risk factor control for a patient who has coronary heart disease diagnosed in the ambulatory setting is optional and based on the results of further diagnostic testing.

## Results

We compared the classification by the computer algorithm to manual record review in 254 patients in the first iteration. After we developed the computer algorithm for the first iteration, we learned that ECG analysis software used by HPMG at that time did not permit searching for abnormalities across patients. Thus, we could not distinguish between STEMIs and nSTEMIs without manual review of the ECGs. However, when we grouped STEMIs and nSTEMIs together, we achieved 94% (60/64) agreement between the computer algorithm and manual review of the cases that were identified as myocardial infarction by the computer algorithm. Agreement in the other categories ranged from 38% for ambulatory presentation to 52% for systolic heart failure; 46 of the cases selected by the computer algorithm did not fit any of the 6 categories by manual record review. Overall agreement on the first iteration was 57% (Cohen’s κ, 0.47; 95% confidence interval [CI], 0.41–0.54) when STEMIs and nSTEMIs were considered as a single category (Table).

We compared classification with the computer algorithm to manual review in 245 patient records for the second iteration. Agreement was 67% (16/24) for STEMI diagnoses and 100% (21/21) for heart failure diagnoses. Agreement for nSTEMI diagnoses was 78% (39/50) and 38% (19/50) for unstable angina. Agreement was 46% (23/50) for ambulatory presentation and 90% (45/50) for chronic disease diagnoses. Overall agreement was 67% (163/245), and Cohen’s κ was 0.61 (95% CI, 0.55–0.68) (Table).

We compared classification with the computer algorithm to manual review in 184 cases for the third iteration. The only disagreement was a single patient diagnosis identified as an nSTEMI by the computer algorithm and systolic heart failure by the reviewers. Overall agreement was greater than 99%, and Cohen’s κ was 0.99 (95% CI, 0.98–1.00).

## Discussion

By using an algorithm that processes only electronic data from the medical records of patients treated for coronary heart disease or heart failure, we were able to replicate the clinician’s diagnosis with a high degree of accuracy. To distinguish between STEMI and nSTEMI, the algorithm uses highly specific diagnostic statements (7) that map to ICD-9-CM, ICD-10, SnoMED CT, and Unified Medical Language System (8). Four-digit ICD-9-CM codes are not detailed enough to make the distinction, because the code 410.9 can represent both STEMI and nSTEMI.

Our study has limitations. The analysis is based on the records of only 1 medical group; other groups may have a different experience, so algorithm performance should be tested before adoption for routine use. The purpose of this study is to determine the best algorithm with which to estimate the number and type of acute events in a population. If the purpose were to identify patients for case management or to possibly change treatment strategies, a final step of manual record review might be indicated.

It appears unlikely that electronic data can be used to distinguish unstable angina pectoris from symptoms of chest pain and dyspnea that are not due to ischemic heart disease. However, we believe that this is not an important task. Given the sensitivity of troponin assays to detect ischemia and the fact that many of the patients who have normal troponin levels while hospitalized with chest pain or dyspnea are already known to have heart disease, it is probably not necessary to identify this class of patients. There is no compelling evidence that angioplasty reduces mortality in patients who have normal troponin levels in association with their chest pain. Proceeding to angiography would still be an option, but in this case angioplasty would be for symptom control, not mortality reduction.

The use of the ECG database to classify patients may deserve further attention. Only 3 of the 16 diagnoses that manual record review confirmed as STEMIs in the second iteration had codes in the ECG database that indicated ST elevation. Conversely, most of the ECGs with ST elevation codes were not associated with acute myocardial infarction. Using a combination of codes that indicates ST elevation followed by the appearance of Q-wave codes may be more fruitful, but if rescue angioplasty prevents the development of Q-waves, even this strategy would not detect STEMIs.

Our effort to classify events without manual record review is not unique. Natural language processing has been used to assess quality of care for acute myocardial infarction (9), and the eMERGE (Electronic Medical Records and Genomics) Network is promoting the sharing of case identification algorithms, mainly for genomics research (10). A systems dynamics simulation program developed by several organizations, including the Centers for Disease Control and Prevention (CDC), can be used to estimate the effect of preventive interventions, but it cannot compare the effect of preventive interventions with treatments of acute events (11).

It may not seem appropriate to accept the clinical diagnosis as the gold standard for acute myocardial infarction. Ideally, criteria would be based entirely on objective evidence. However, several points must be acknowledged. First, our goal with the project is to develop a method by which the clinician’s diagnosis can be identified from electronic media with the granularity that is necessary to assess whether the patient received all of the evidence-based care that was indicated. We have shown that this is possible. Whether the clinician made the correct diagnosis is a different question. Second, all criteria depend to some extent on diagnostic coding, interpretation of laboratory data collected, and subjective interpretation of patient symptoms. Third, the development of a universal definition of myocardial infarction (12) means that, at least for centers where cardiologists practice, there is agreement about the meaning behind the diagnostic codes that are being assigned to patients’ events.

In 2007 the American Heart Association issued a scientific statement outlining the essential features of a surveillance system that would support the prevention and management of heart disease and stroke (13). Among other components, the statement called for the design and conduct of nationally representative surveillance programs that oversample ethnic subgroups and certain counties. The statement also declared that mechanisms should be developed to enable linkage between health care data systems, national surveillance programs, and electronic health records. The authors of the report acknowledged that the cost of these endeavors would be barriers to implementation. However, the changing health care landscape could markedly reduce the cost of these programs. If universal coverage is implemented and other electronic health record systems also have the capability to identify heart disease prevalence and gaps in evidence-based care, an opportunity to use the electronic health record as a public health surveillance tool at minimal cost will have been created.

## Figures and Tables

**Table:** 

**1. The activity supported the learning objectives.**				
**Strongly Disagree**				**Strongly Agree**
1	2	3	4	5
**2. The material was organized clearly for learning to occur.**				
**Strongly Disagree**				**Strongly Agree**
1	2	3	4	5
**3. The content learned from this activity will impact my practice.**				
**Strongly Disagree**				**Strongly Agree**
1	2	3	4	5
**4. The activity was presented objectively and free of commercial bias.**				
**Strongly Disagree**				**Strongly Agree**
1	2	3	4	5

## Post-Test Information

To obtain credit, you should first read the journal article. After reading the article, you should be able to answer the following, related, multiple-choice questions. To complete the questions (with a minimum 70% passing score) and earn continuing medical education (CME) credit, please go to http://www.medscape.org/journal/pcd. Credit cannot be obtained for tests completed on paper, although you may use the worksheet below to keep a record of your answers. You must be a registered user on Medscape.org. If you are not registered on Medscape.org, please click on the "Register" link on the right hand side of the website to register. Only one answer is correct for each question. Once you successfully answer all post-test questions you will be able to view and/or print your certificate. For questions regarding the content of this activity, contact the accredited provider, CME@medscape.net. For technical assistance, contact CME@webmd.net. American Medical Association's Physician's Recognition Award (AMA PRA) credits are accepted in the US as evidence of participation in CME activities. For further information on this award, please refer to http://www.ama-assn.org/ama/pub/category/2922.html. The AMA has determined that physicians not licensed in the US who participate in this CME activity are eligible for **AMA PRA Category 1 Credits™**. Through agreements that the AMA has made with agencies in some countries, AMA PRA credit may be acceptable as evidence of participation in CME activities. If you are not licensed in the US, please complete the questions online, print the AMA PRA CME credit certificate and present it to your national medical association for review.

## Post-Test Questions

### Article Title: An Algorithm That Identifies Coronary and Heart Failure Events in the Electronic Health Record

**CME Questions**

1. You are part of a task force trying to implement the 2007 American Heart Association recommendations for implementation of a surveillance system for cardiovascular disease. Which of the following statements regarding these recommendations is most accurate?
  - The focus of the surveillance should be limited to local populations only
  - All counties and ethnic subgroups should be represented equally in the sample
  - Systems should be in place to link healthcare data systems with the surveillance program
  - Cost should not be a factor in the implementation of a successful surveillance program
2. You want to set up the cardiovascular disease surveillance program using electronic health records. Which of the following statements regarding the accuracy of electronic vs manual records in the current study is most accurate?
  - Even basic electronic health records correlated well with manual records
  - Only electrocardiogram (ECG) data helped to correlate electronic health records with manual records
  - Agreement between electronic and manual health records was largely dependent upon modifications to the original iteration
  - There was no substantial agreement between manual and electronic health records in any study analysis
3. Your electronic health record contains an ECG analysis system. What should you consider regarding ECG data and myocardial infarctions in the current study?
  - The electronic ECG analysis was only effective in differentiating ST-segment elevation myocardial infarctions (STEMIs) from non-STEMIs (NSTEMIs)
  - The ECG system was effective in achieving agreement in the overall diagnosis of myocardial infarction
  - The ECG system effectively achieved agreement regarding myocardial infarction and the presence of ST elevation
  - The first iteration of the current system failed to identify myocardial infarction in all analyses
4. What else should you consider regarding electronic data in the current study?
  - Adding a more specific ICD-9 code with a fifth digit improved the accuracy of the electronic health record
  - The electronic health record outperformed the manual record in differentiating causes of chest pain
  - Most ECGs in the database with ST elevation were associated with diagnosis of acute myocardial infarction
  - STEMIs were identified to similar degrees in the manual and electronic health records

**Evaluation**
